# Comparing serum protein levels can aid in differentiating HPV-negative and -positive oropharyngeal squamous cell carcinoma patients

**DOI:** 10.1371/journal.pone.0233974

**Published:** 2020-06-15

**Authors:** Amy Dickinson, Mayank Saraswat, Stina Syrjänen, Tiialotta Tohmola, Robert Silén, Reija Randén-Brady, Timo Carpén, Jaana Hagström, Caj Haglund, Petri Mattila, Antti Mäkitie, Sakari Joenväärä, Suvi Silén

**Affiliations:** 1 Department of Otorhinolaryngology—Head and Neck Surgery, University of Helsinki and Helsinki University Hospital, Helsinki, Finland; 2 Research Program in Systems Oncology, Faculty of Medicine, University of Helsinki, Helsinki, Finland; 3 Transplantation Laboratory, Haartman Institute, University of Helsinki, Helsinki, Finland; 4 HUSLAB, Helsinki University Hospital, Helsinki, Finland; 5 Department of Oral Pathology and Oral Radiology, University of Turku, Turku, Finland; 6 Department of Pathology, Turku University Hospital, Turku, Finland; 7 Department of Pathology, University of Helsinki and Helsinki University Hospital, Helsinki, Finland; 8 Department of Surgery, University of Helsinki and Helsinki, University Hospital Helsinki, Helsinki, Finland; 9 Research Programs Unit, Translational Cancer Medicine, University of Helsinki, Helsinki, Finland; 10 Division of Ear, Nose and Throat Diseases, Department of Clinical Sciences, Intervention and Technology, Karolinska Institutet and Karolinska University Hospital, Stockholm, Sweden; 11 Department of Biosciences and Nutrition, Karolinska Institutet, Stockholm, Sweden; University of Wisconsin, UNITED STATES

## Abstract

**Background:**

The surrogate immunohistochemical marker, p16^INK4a^, is used in clinical practice to determine the high-risk human papillomavirus (HPV) status of oropharyngeal squamous cell carcinomas (OPSCC). With a specificity of 83%, this will misclassify some patients compared with direct HPV testing. Patients who are p16^INK4a^-positive but HPV DNA-negative, or RNA-negative, may be unsuitable for treatment de-escalation aimed at reducing treatment-related side effects. We aimed to identify cost-effective serum markers to improve decision making for patients at risk of misclassification by p16^INK4a^ alone.

**Methods:**

Serum proteins from pre-treatment samples of 36 patients with OPSCC were identified and quantified using label-free mass spectrometry-based proteomics. HPV-status was determined using p16^INK4a^/HPV DNA and E6/E7 mRNA. Serum protein expressions were compared between groups of patients according to HPV status, using the unpaired t-test with a Benjamini-Hochberg correction. ROC curves (AUC) were calculated with SPSS (v25).

**Results:**

Of 174 serum proteins identified, complement component C7 (C7), apolipoprotein F (ApoF) and galectin-3-Binding Protein (LGALS3BP) significantly differed between HPV-positive and -negative tumors (AUC ranging from 0.84–0.87). ApoF levels were more than twice as high in the E6/E7 mRNA HPV-positive group than HPV-negative.

**Conclusions:**

Serum C7, ApoF and LGALS3BP levels discriminate between HPV-positive and HPV-negative OPSCC. Further studies are needed to validate these host immunity-related proteins as markers for HPV-associated OPSCC.

## Introduction

Human papilloma virus (HPV) is an established aetiological factor for oropharyngeal squamous cell carcinoma (OPSCC), with nearly 90% of HPV-positive OPSCCs being caused by the high-risk genotype HPV16 [1]. The incidence of OPSCC has been rising in Western countries, mainly due to the increase in HPV-positive disease. HPV prevalence in OPSCC is globally variable [1–5], and it also relates to the methods used for HPV detection as recently shown in a comprehensive assessment suggesting that only 25% of all OPSCCs are HPV positive worldwide [6]. Moreover, HPV-positive OPSCCs have a different pattern of histopathological features and clinical behavior compared with HPV-negative OPSCCs [7, 8]. They tend to occur in younger patients, and although HPV-positive tumors are more likely to present with nodal metastases, they generally have a favorable prognosis compared with alcohol- and smoking-related, non-HPV OPSCCs [9–11]. Furthermore, patients with HPV-related OPSCC may benefit from de-escalated treatment regimens, intended to minimize toxicity [12].

The difference in the tumor behavior is reflected in the latest WHO classification of head and neck tumors, in which HPV-positive and HPV-negative OPSCCs are classified as distinct clinical entities, using the overexpression of p16^INK4a^ as a surrogate immunohistochemical marker for HPV status [13, 14]. According to a meta-analysis by Prigge et al., p16^INK4a^ testing has a sensitivity of 94% [15]. CDKN2A, the gene encoding p16^INK4a^, is tumor suppressor gene through the inhibition of the catalytic activity of the cyclin D1/CDK4/6 complex. The cyclin D1/CDK4/6 complex phosphorylates the retinoblastoma protein (pRb), resulting in the release of the transcription factor E2F and the initiation of cell-cycle progression. Conversely, the pRb/E2F repressor complex inhibits transcription of several genes, including CDKN2A [16]. By inactivating pRb, HPV-E7 releases the CDKN2A gene from its transcriptional inhibition that results in p16^INK4a^ overexpression, and it accumulates within the cells [17]. Despite its high sensitivity, p16^INK4a^ has a specificity of 83% [15] as it is involved in other cellular processes such as cell senescence [18]. Frequent HPV-independent overexpression of p16^Ink4a^ in head and neck carcinomas has been explained by mutation or amplification of CDKN2A or by mutations in RB1 or histone H3 lysine 36 methyltransferase genes [19].

The accepted gold standard method for the detection of HPV with an aetiological role in OPSCC is testing for E6/E7 mRNA, based on the assumption that E6 and E7 oncogenes are persistently transcribed in HPV-associated carcinomas [20]. However, E6/E7 mRNA detection is methodologically more challenging, thus limiting its use in clinical routine [15]. The meta-analysis by Prigge et al. compared p16^INK4a^ and HPV DNA detection methods, alone and combined, and suggested that p16^INK4a^ immunohistochemistry (IHC) combined with HPV DNA PCR testing has an enhanced specificity (96%) while maintaining high sensitivity (93%) [15]. Mena et al. reported recently that the combination of HPV DNA and p16^INK4a^ positivity also provided the strongest diagnostic accuracy as well as prognostic value in OPSCC [21]. Currently, the p16^INK4a^ surrogate marker is used in the clinic to determine whether the OPSCC is HPV-positive or HPV-negative. However, there is a specific subgroup of OPSCCs, which are p16^INK4a^ -positive but negative for HPV DNA, and these have been reported to have distinctive clinical and morphological features, and a worse prognosis and increased risk of distant metastases than those positive for both p16^Ink4a^ and HPV DNA [7, 22, 23].

At present, there is a consistent misclassification of some patients with OPSCC, as no method for determining the HPV status of an OPSCC is completely accurate. Therefore, we set about identifying serum proteins that could be simply and cost-effectively tested, to improve the detection of mis-classified patients.

To this end, we have used a label-free quantitative mass spectrometry methodology to identify the serum proteome in patients with OPSCC, comparing the protein expression levels between patients according to p16^INK4a^, DNA and E6/E7 mRNA statuses: E6/E7 mRNA -positive vs -negative, and p16^INK4a^/DNA -positive vs -negative. The aim of this was to identify serum proteins that could discriminate between the p16^INK4a^+ OPSCCs that are HPV-associated, and those that are p16^INK4a^+ but HPV-independent.

## Materials and methods

### Workflow and data availability

**Fig 1:**
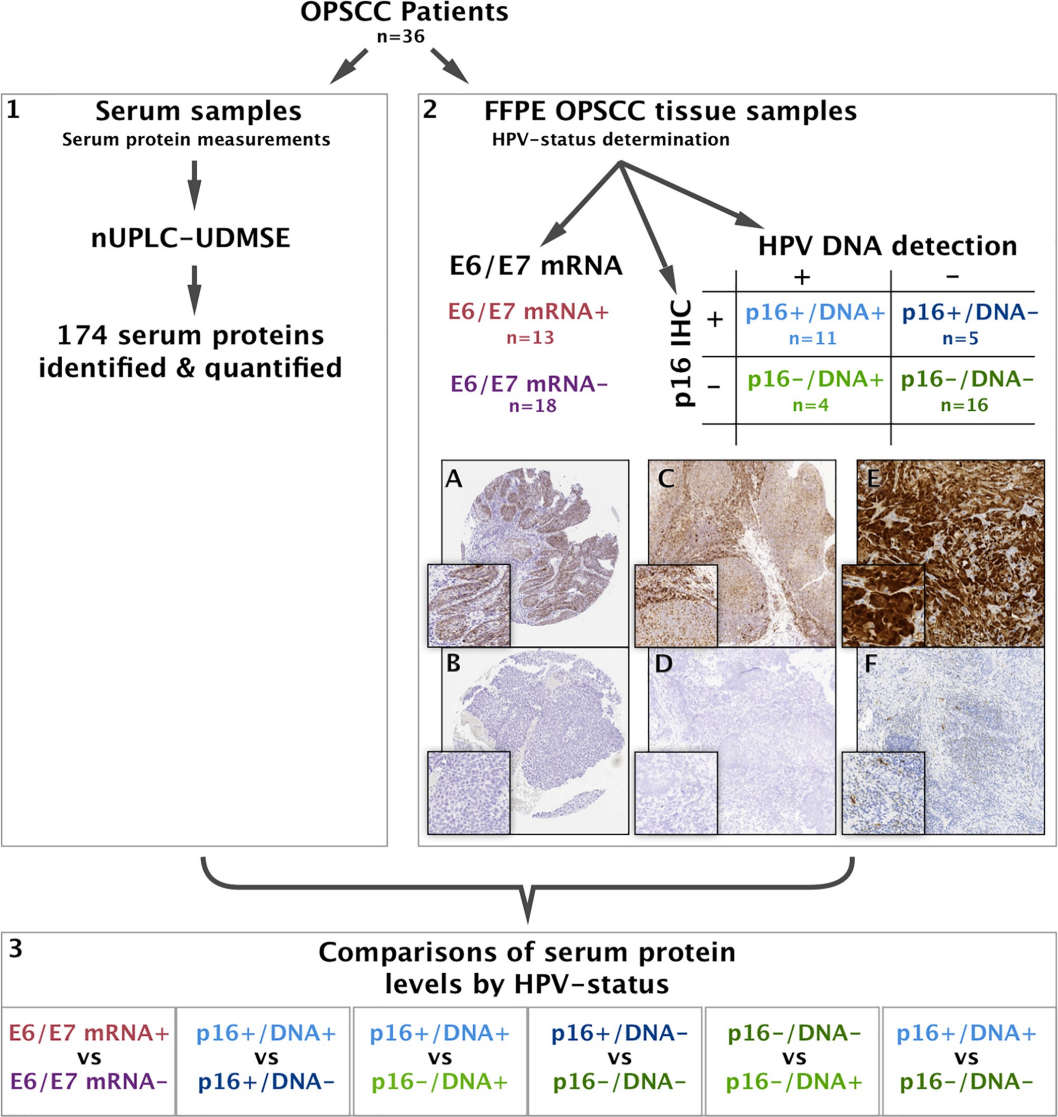
Workflow. Serum samples and FFPE samples were analysed from patients with OPSCC. 1. Serum proteins were identified and quantified using nano ultra-performance liquid chromatography—ultra definition mass spectrometry. 2. From formalin-fixed paraffin-embedded samples, HPV-status was determined using the following methods: E6/E7 mRNA was detected using RNAScope, to detect transcriptionally active HPV; p16 was detected using p16INK immunohistochemistry; HPV DNA was detected using PCR. 2A shows a representative TMA spot positive for E6/E7 mRNA; 2B shows a representative TMA spot negative for E6/E7 mRNA; 2C shows the positive PPIB probe for RNA analysis; 2D shows DAPB negative probe for the RNA analysis; 2E shows a representative p16 positive FFPE slide; 2F shows a representative p16 negative FFPE slide. Magnification is 100x and 200x. 3. Combining the serum proteomic data with the determined HPV-status, we compared the protein abundance between the groups.

The overall workflow is shown in **Fig 1**.

It is work noting that the serum proteomics data is from an overarching serum proteomics mass spectrometry project and overlaps with another cohort that has previously been published by Tuhkuri et al. [24], Tuhkuri et al. compared serum proteins from early-stage OPSCC patients with controls and also separately compared early-stage p16+ and p16- OPSCCs with each other. In comparison with that study, we have used all stages of tumors, and have used additional methods to determine HPV-positivity. The mass spectrometry data for the samples used in both overlapping projects are available in the same overall project in the ProteomeXchange Consortium via the PRIDE partner repository with the data set identifier PXD008445 [25]. **S1 Table** explains which data files were used in this paper.

The data from HPV DNA detection, p16 and RNA scope on these patients has also been published elsewhere by Randen-Brady et al. [26] (the PM II cohort).

### Patients and serum samples

Pre-treatment serum samples, as well as formalin-fixed paraffin-embedded (FFPE) samples, were collected from 36 patients with both of these samples available, diagnosed with OPSCC between the years 2012 and 2016 at the Department of Otorhinolaryngology–Head and Neck Surgery, Helsinki University Hospital, Helsinki, Finland. Clinical data were collected from patient records. The UICC (Union for International Cancer Control) 8^th^ edition TNM classification [14] was used to evaluate tumor stage.

After being allowed to clot at room temperature, blood samples were centrifuged at 4°C at 1000xg for 10 minutes in order to separate the serum. Sera were aliquoted and stored at -70°C, until they were all assayed at the same time.

The study plan was approved by the Helsinki University Hospital’s institutional Research Ethics Board (DNr. 51/13/03/02/2013). Informed written consent was obtained from all patients. The study was performed in accordance with the latest version of the Declaration of Helsinki.

### Tissue p16^INK4a^ detection

p16^INK4a^ was detected immunohistochemically on FFPE tissue samples on individual slides as previously described [26, 27]. Briefly, after deparaffinization and rehydration a pre-treatment-module (Lab Vision Corp., UK Ltd., UK) was used for treating the tissue slides in Tris-HCl buffer (pH 8.5). Endogenous peroxidase was blocked using 0.3% Dako REAL Peroxidase-Blocking Solution. The Dako REAL Envision detection system (Agilent Technologies Inc, CA, USA) was used according to manufacturer’s instructions, and the primary antibody was the “ready-to-use” monoclonal mouse anti-human p16^INK4a^ (9517 CINtec Histology Kit, MTM laboratories, Germany). If over 70% of tumor cells were strongly immunopositive for p16^INK4a^, the tumor was defined as p16^INK4a^ positive [28]. As a positive control a known p16+ tonsil cancer was used, and negative controls were incubated without the primary antibody.

### Tissue HPV DNA detection

An HPV PCR assay was used to detect HPV DNA in the tissue samples. Firstly, DNA was extracted from 4 to 10 μm diameter sections cut from formalin fixed paraffin embedded (FFPE) tissue sections using the high salt method as described earlier [29]. For HPV genotyping, Multiplex HPV Genotyping Kit® (DiaMex GmbH, Germany) was used as described earlier [22, 26, 27]. Primer Set 1 contains all HPV primers: 9 biotinylated forward and 3 reverse primers for amplifying the HPV types under investigation. Primer Set 2 (DNA quality control primers) contains primers for the amplification of a ß-globin gene fragment to verify the amount and the quality of human genomic sample DNA. Every eighth sample was a negative control without any genomic DNA to confirm the absence of contamination in the amplification reactions [30]. The labeled hybrids were analyzed with a Luminex LX-100 analyzer (Bio-Plex 200 System, Bio-Rad Laboratories, Hercules, USA). The Multiplex HPV Genotyping Kit detects 24 LR- and HR-HPV-genotypes as follows: LR-HPV6, 11, 42, 43, 44, and 70; and HR-HPV16, 18, 26, 31, 33, 35, 39, 45, 51, 52, 53, 56, 58, 59, 66, 68, 73 and 82.

### In situ hybridization for high-risk HPV E6/E7 mRNA

In situ hybridization (ISH) for high risk HPV E6/E7 mRNA was performed manually using the RNAscope® 2.5 HD Reagent kit (Advanced Cell Diagnostics, Inc., Hayward, CA) according to the manufacturer´s protocol as described also in detail in our recent papers [26, 31]. Five-μm formalin-fixed and paraffin-embedded tissue microarray (TMA) sections were incubated for 1 hour at 58°C to fix the samples onto slides. After deparaffinization, the sections were pre-treated with hydrogen peroxidase (RNAscope® Hydrogen Peroxide) for 10 minutes at RT. Target retrieval was performed (RNAscope® Target Retrieval Reagents) for 15 minutes at 100°C. The sections underwent protease treatment (RNAscope® Protease Plus) for 30 minutes at 40°C in the hybridization oven followed by hybridization with the HR HPV 18 cocktail probe (RNAscope®) for genotypes 16, 18, 26, 31, 33, 35, 39, 45, 51, 52, 53, 56, 58, 59, 66, 68, 73 and 82 for 2 hours at 40°C in the hybridization oven. Pre-amplifiers and amplifiers were hybridized consecutively, accompanied by chromogenic signal detection with 3,3’-diaminobenzidine (DAB). Finally, the slides were counterstained with hematoxylin. An endogenous housekeeping gene HS-PPIB (RNAscope®) probe was used as a positive control, and a bacterial gene DapB, diaminopimelate (RNAscope®) probe used as a negative control. The staining was examined using a qualitative scoring system: punctate brown, granular nuclear and cytoplasmic dots were either observed or not in the individual TMA spots. 1–3 brown dots or more/tumor cell was recorded as a positive staining result.

### Serum treatment and protein digestion

These methods have been described previously in detail [32] and also by Tuhkuri et al. [24]. Briefly, the top 12 proteins were depleted from thawed serum samples, which increases the dynamic range of the lower abundance proteins. Total protein levels in each sample were measured using a bicinchoninic acid assay kit (Pierce, Thermo Scientific, Rockform, IL). The samples, amounting to 350μg of total protein were dried in a speed vacuum then dissolved in a urea-containing digestion buffer. They were digested overnight using trypsin (Promega, Madison, WI), having been reduced and alkylated. Then they were purified using C18 columns (Thermo Scientific, Rockform, IL), and resuspended in a solution of 0.1% formic acid, which contained 12.5gmol/μl of Hi3 E.coli peptides (Waters Corporation, MA). All described procedures were performed according to the manufacturer’s instructions, where applicable.

### Liquid chromatography–ultra-definition mass spectrometry (LC-UDMS^E^)

This was performed as previously described in detail [24] and also by Tuhkuri et al. [24]. Four μL samples corresponding to approximately 1.4μg of total protein, were injected into the nano ultra-performance liquid chromatography (UPLC) system (Waters Corporation, MA) [33]. The TRIZAIC nanoTile 85μm x 100mm HSS-T3u wTRAP was applied as a separating device before mass spectrometry (MS). Buffers were made up using UPLC-grade chemicals (Sigma-Aldrich, MO). Buffer A: 0.1% formic acid in water, Buffer B: 0.1% formic acid in acetonitrile. Samples were trapped and washed with a mixture of Buffer A and B, initially using 1% Buffer B, with a gradient of Buffer A/Buffer B occurring over 90 minutes for each sample, to elute specific peptides. Data were acquired with UDMSE with Synapt G2-S UDMS (Waters Corporation, MA) including ion mobility spectroscopy (IMS). The data range was 100–2000m/z, scan time 1s, IMS wave velocity 650ms−1 and collision energy ramped in trap between 20 and 60V. Calibration was performed using Glu1-fibrinopeptide B MS2 fragments, and Glu1-fibrinopeptide B precursor ion was used during the acquisitions as a lock mass. 10% of the samples were acquired as triplicates to validate the results, and further analysis was done with Progenesis QI for Proteomics software (Nonlinear Dynamics, Newcastle, UK). The mass spectrometry proteomics data have been deposited into the ProteomeXchange Consortium via the PRIDE partner repository with the data set identifier PXD008445 [25]. **S1 Table** explains which data files were used.

### Mass spectrometry data analysis

The data analysis has been described previously in detail [34]. Briefly, Progenesis QI for proteomics software (v3, Nonlinear Dynamics) was used for raw data processing, using default parameters for peak picking and alignment. Peptide identification was run against Uniprot human FASTA sequences (UniprotKB Release 2015_09, 20205 sequence entries), and label-free protein quantification performed using the Hi-N method (Protein Lynx Global Server) [35], whereby the samples were spiked with 12.5fmol/μL of CLPB_ECOLI (P63285, ClpB protein) peptides (Hi3 Escherichia Coli Standard, Waters). The peptide identification parameters were: fixed modification of cysteine (carbamidomethyl) and variable modification of methionine (oxidation). The peptide error tolerance was set to a maximum of 10ppm, the false discovery rate limited to less than 2% and default values (in Progenesis QI for Proteomics) were used for the rest of the parameters.

### Comparisons

The following comparisons between the samples’ protein abundances were performed:

E6/E7 mRNA+ (n = 13) vs E6/E7 mRNA- (n = 18)
p16^INK4a^-/DNA- (n = 16) vs p16^INK4a^+/DNA+ (n = 11)
p16^INK4a^+/DNA- (n = 5) vs p16^INK4a^-/DNA- (n = 16)
p16^INK4a^-/DNA+ (n = 4) vs p16^INK4a^-/DNA- (n = 16)
p16^INK4a^-/DNA+ (n = 4) vs p16^INK4a^+/DNA+ (n = 11)
p16^INK4a^+/DNA- (n = 5) vs p16^INK4a^+/DNA+ (n = 11)

### Statistical analysis

The data were imported into EZinfo 3.0.3.0 (Release date Dec 02, 2014, Umetrics, Sweden), a statistical package that works with Progenesis QI for proteomics. The quantified proteins in the comparisons were compared using an unpaired t-test on a protein-by-protein basis, with a p-value of 0.05 being the cut-off for statistical significance. The Benjamini-Hochberg multiple testing correction was performed, with an FDR cut-off of 0.1. ROC curves were calculated using SPSS (version 25).

## Results

### Patient demographics and tumor characteristics

Serum from 36 patients with newly diagnosed OPSCC was analyzed. Clinical parameters are shown in **S2 Table.** All samples that were positive for HPV DNA contained HPV16 DNA, except for one sample that was HPV18 positive; this tumor was p16^INK4a^- but E6/E7 mRNA+.

Representative images of p16+ and p16-, E6/E7 mRNA+ and E6/E7 mRNA- are shown in Fig 1 as well as in **S1 Fig**. HPV DNA results are shown in **S3 Table**.

### Proteomics

A total of 174 serum proteins with 2 or more unique peptides were identified and quantified by mass spectrometry. **Table 1** shows the significant proteins after multiple testing correction, between HPV-positive and HPV-negative as tested using E6/E7 mRNA and p16^INK4a^/DNA, respectively. The other comparisons, which included samples with discordant p16^INK4a^/DNA yielded no significant proteins. **S4 Table** details all of the identified proteins for each comparison.

**Table 1:** Serum proteins differing between patients with a) E6/E7 mRNA -positive and -negative OPSCCs and b) p16^INK4a^/DNA -positive and -negative OPSCCs.

**E6/E7 mRNA+ vs mRNA- proteins**					
Accession	Protein name	p-value	FDR	FC (mRNA-/mRNA+)	ROC
P10643	Complement component C7	0.000677	0.058859	1.37	0.87
Q13790	Apolipoprotein F	0.000542	0.058859	0.41	0.84
**p16**^**INK4a**^ **+/DNA+ vs p16**^**INK4a**^**-/DNA-**					
Accession	Protein name	p-value	FDR	FC (p16^INK4a^-DNA-/ p16^INK4a^+ DNA+)	ROC
P10643	Complement component C7	0.000911	0.079284	1.42	0.86
Q08380	Galactin-3-Binding Protein	0.000879	0.079284	1.37	0.86

P-values were calculated using the unpaired t-test. These proteins remained significant following multiple-testing correction, with FDR set to 0.1. FC–fold change; ROC–receiver operating characteristic; abundance–average normalized abundance.

## Discussion

In this study, using nUPLC-UDMS^E^, we aimed to identify serum proteins that could be easily and economically implemented to make a differential diagnosis between HPV-associated and HPV-independent OPSCCs. This approach would improve the detection of patients whose OPSCCs are mis-classified with regards to HPV status by p16^INK4a^ testing. For this, we compared the proteomic differences using different proxies for HPV: E6/E7 mRNA, and p16^INK4a^ combined with HPV DNA. We identified three proteins (ApoF, Galactin-3-Binding protein (LGALS3BP) and complement component C7 (C7) that differentiate between HPV+ and HPV- tumors. C7 was the only protein that differentiated between HPV+ and HPV- tumors using either p16^INK4a^/DNA or E6/E7 mRNA as HPV detection methods.

ApoF is a sialoglycoprotein, also known as “lipid transfer inhibitor protein”, and is an inhibitor of cholesterol ester transferase that binds to LDL, and regulates cholesterol transport [36]. Lipid metabolic reprogramming is a known hallmark of cancer, and it has been shown that inhibition of cholesterol production with statins in head and neck cancers is associated with improved disease-specific survival [37]. In animal studies, ApoF has also been associated with immune and inflammation responses [38]. Transcription of interferon alpha (IFNα) responsive genes was shown to be impaired in the ApoF knockout mice and attributable also to hypomorphic expression of Stat2 [39]. These genes and the type I IFN pathway are of major importance also in HPV infections as they modulate the transcription HPV E6 and E7 oncogenes and viral immune evasion [40]. Thus, the increased levels of ApoF in sera might be interpreted as being due to general metabolic reprogramming of malignant invasive cells, or due to reprograming of the malignant cells and the surrounding stromal reaction by HPV *per se*.

We identified significantly different expression of Complement component C7 using either the E6/E7 mRNA proxy or the p16^INK4a^/DNA proxy (**Table 1**). C7 is a critical component of the terminal pathway of complement activation. C7, along with complement components 6, 8, and 9, is involved in membrane attack complex (MAC) formation [41]. Sublytic MACs, which form when the nucleated cells are not unequivocally identified as non-self can activate a few oncogenic pathways such as the mitogen-activated protein kinase (MAPK) family, extracellular regulated protein kinases (ERKs), p38 MAPK, the phosphatidylinositol 3-kinase (PI3K) pathway and Ras [42]. These pathways are of importance in HPV-infected cells in the progression toward malignancy [43].

Thus, similarly as with ApoF, these increased serum concentrations of C7 may result from local upregulation of complement genes in primary tumor sites, mainly by endothelial cells and cells participating in immune responses i.e. the integral component of both the premetastatic tumor niche the tumor microenvironment. Elevated serum levels of C7 have been linked to a better prognosis in ovarian and non-small cell lung cancer (NSCLC). Additionally, overexpression of C7 was shown to inhibit the colony formation of NSCLC cells, which indicates that C7 might be a potential tumor suppressor [44].

LGALS3BP is a secreted glycoprotein that was originally identified during investigations into different malignancies. Furthermore, the expression of LGALS3BP is upregulated in both chronic and acute viral infections, perhaps due to its induction by a variety of molecules that “either mimic or are characteristic for an ongoing inflammation and microbial infection, such as IFN-*α*, IFN-*β*, IFN-*γ*, TNF-*α*, poly(I:C), dsRNA, and dsDNA”, as thoroughly reviewed by Loimaranta et al. [45]. As one member of the scavenger receptor cysteine-rich (SRCR) domain-containing protein family, which are implicated in both self-nonself discrimination and innate immunity-related proteins [45]. Thus, it was not unexpected to identify that LGALS3BP sera levels were associated with an HPV associated OPSCC. In the future, it would be important to study whether high sera levels of LGALS3BP could be associated with other viral associated malignancies such as EBV-associated nasopharyngeal carcinomas and lymphomas. Higher serum LGALS3BP levels have been linked to poor prognosis and progression in various cancers [46–49].

The main strength of the study is that, to our knowledge, this the first time that the serum proteomes have been assessed and compared in OPSCC patients with different p16^INK4a^ and HPV statuses, to identify possible serum biomarkers that can improve classification of patients who would be misclassified when using p16^INK4a^ or HPV DNA testing.

Importantly, all the identified three proteins could be linked also immunity and inflammatory responses also of importance for HPV *per se*. However, it remains to seen whether these proteins are specific enough for HPV or whether they are reflecting also other viral associated malignancies.

Limitations of this study include small group sizes. As a consequence of this, and insufficient smoking data, we were unable to compare the proteomic signatures of those with a <10 year smoking and >10 year smoking history, and different disease stages, which are known to affect the prognosis of these tumors [50, 51].

## Conclusions

We have identified three interesting serum proteins with the potential to improve stratification of OPSCC patients. Serum levels of C7, APOF and LGALS3BP differentiate between HPV-positive and HPV-negative OPSCC. Further research is warranted to confirm these findings on a larger cohort.

## Supporting information

S1 Table Details of files used from dataset PXD008445. (XLSX)

S2 Table Clinical parameters of the patients. (DOCX)

S3 Table HPV DNA PCR results. (XLSX)

S4 Table All serum proteins with 2 or more significant proteins, with statistical information. (XLSX)

S1 Fig Large versions of the p16-positive, p16-negative, E6/E7 mRNA+, E6/E7 mRNA- and RNAScope -positive and -negative probes, also shown as a small version in Fig 1. (EPS)
