# Supplementary material for: Comparing serum protein levels can aid in differentiating HPV-negative and -positive oropharyngeal squamous cell carcinoma patients
Source: PLoS One. 2020 Jun 15;15(6):e0233974. doi: 10.1371/journal.pone.0233974 (PMC7295232; doi:10.1371/journal.pone.0233974)
Supplement: S2 Table — (DOCX) [file pone.0233974.s002.docx]

| **Patient number** | **Gender** | **Age at diagnosis** | **Tumour location** | **TNM^a^** | **HPV DNA and subtype** | **p16** | **E6/E7 mRNA** |
| --- | --- | --- | --- | --- | --- | --- | --- |
| 1 | female | 64 | Tongue base | T4aN2c | + 16 | - | - |
| 2 | male | 75.5 | Tonsil | T4bN2b | + 16 | - | - |
| 3 | female | 57.7 | Pharynx back wall | T2N0 | + 16 | - | - |
| 4 | male | 71.8 | Tongue base | T4aN2c | + 18 | - | + |
| 5 | female | 71 | Tongue base | T4aN2a | - | - | N/A |
| 6 | female | 62.4 | Tonsil | T2N0 | - | - | N/A |
| 7 | female | 63.9 | Pharynx back wall | T4bN1 | - | - | - |
| 8 | female | 71.4 | Soft palate | T2N0 | - | - | - |
| 9 | male | 69.5 | Tongue base | T2N0 | - | - | - |
| 10 | male | 60.5 | Tonsil | T2N0 | - | - | - |
| 11 | female | 66.4 | Tonsil | T4bN2c | - | - | - |
| 12 | female | 62.8 | Tongue base | T1N0 | - | - | - |
| 13 | male | 58.4 | Tongue base | T4aN0 | - | - | N/A |
| 14 | male | 60.1 | Soft palate | T4aN2b | - | - | - |
| 15 | male | 57.2 | Soft palate | T1N0 | - | - | - |
| 16 | male | 57.6 | Soft palate | T2N0 | - | - | - |
| 17 | female | 65.8 | Tonsil | T4aN2b | - | - | - |
| 18 | male | 59.2 | Soft palate | T4aN2c | - | - | + |
| 19 | female | 64.1 | Tongue base | T4aN0 | - | - | - |
| 20 | male | 49.2 | Soft palate | T2N0 | - | - | - |
| 21 | male | 36.6 | Tongue base | T1N2b | - | + | N/A |
| 22 | female | 63.3 | Tonsil | T4aN1 | - | + | N/A |
| 23 | male | 78.4 | Soft palate | T1N2b | - | + | - |
| 24 | male | 84.7 | Tongue base | T4aN2b | - | + | - |
| 25 | female | 55.3 | Pharynx back wall | T4aN0 | - | + | - |
| 26 | male | 55.6 | Tongue base | T4aN2b | + 16 | + | + |
| 27 | male | 58.4 | Tongue base | T4aN1 | + 16 | + | + |
| 28 | male | 63.5 | Tongue base | T4aN0 | + 16 | + | + |
| 29 | male | 66.5 | Tongue base | T4aN2b | + 16 | + | + |
| 30 | male | 67.8 | Tonsil | T4aN2b | + 16 | + | + |
| 31 | male | 59.6 | Soft palate | T2N0 | + 16 | + | + |
| 32 | female | 60.3 | Tonsil | T1N1 | + 16 | + | + |
| 33 | male | 72.5 | Tongue base | T4aN1 | + 16 | + | + |
| 34 | male | 58.7 | Tongue base | T1N2b | + 16 | + | + |
| 35 | male | 54.4 | Tongue base | T4aN2b | + 16 | + | + |
| 36 | female | 61.2 | Tonsil | T2N0 | + 16 | + | + |
|  |  |  |  | ^a^All patients are M0 | | | |
